# Shoulder injury related to COVID-19 vaccine administration: a case report

**DOI:** 10.1016/j.xrrt.2021.10.005

**Published:** 2021-12-04

**Authors:** Benjamin R. Wharton, Kent C. Doan, Michelle L. Wolcott

**Affiliations:** University of Colorado School of Medicine, Department of Orthopedics, University of Colorado, Aurora CO, USA

**Keywords:** COVID-19, SIRVA, Shoulder injury, Injection, Vaccine

With the rollout of the most ambitious worldwide vaccine initiative of all time to combat the COVID-19 pandemic, the medical community has been faced with more challenges than ever before. Vaccine hesitancy has been among one of the leading threats to the global vaccination effort,^10^ and this is presumably due to concerns with vaccine side effects. As side effects are encountered, the health care community has a duty to quickly disseminate these risks to try and mitigate their potential harms. One of the most common side effects of all intramuscular vaccines is a transient local inflammatory reaction, which is maintained in the novel COVID-19 vaccination.^10^ Less common, however, are reactions known as shoulder injury related to vaccine administration (SIRVA), which have only recently been described in mRNA vaccines. We will be presenting a case of SIRVA in a young healthy health care worker as a result of inappropriate site of administration of the Moderna vaccine.

## Case description

The patient is a healthy, 31-year-old right-handed man who works as a full-time health care employee weighing 83.9 kg with a body mass index of 26.6. He presented with left shoulder pain for 2.5 weeks after second administration of the COVID-19 vaccine. He endorses past shoulder injuries bilaterally including right rotator cuff tendonitis about four years prior. He experienced an acute onset of left shoulder pain, weakness, and stiffness after receiving his second dose of the Moderna COVID-19 vaccine 2.5 weeks prior. On injection, he noted the location of the injection site seemed unusually proximal in the deltoid, just distal to his acromion. He had experienced normal postvaccination symptoms including erythema, pain, and induration initially over the first 24 hours. This progressed with continued weakness and pain after the one-day mark, eventually limiting his ability to use his left hand for his job with no improvement over the next 2.5 weeks. He denied any type of neurologic deficits, but was restricted by shoulder stiffness and overall pain. The patient described his pain and stiffness as constant. The symptoms bothered him at night, with exertion, and at rest. They were both localized to the lateral shoulder and were primarily noted during movements of shoulder abduction and flexion. The patient was limited in his ability to work. His only previous treatment included anti-inflammatories. Overall, the problem significantly impacted his quality of life.

When examining the left shoulder, there was no ecchymosis, nor was there any discernable atrophy. There was mild diffuse tenderness to palpation. The patient's active range of motion with abduction and forward elevation was limited to 80 degrees secondary to pain; however, his passive motion was preserved. He did also have subacromial impingement signs with a positive Hawkins test. Both the patient’s supraspinatus empty can test and his infraspinatus external rotation test yielded a strength score of four with notable pain in the left shoulder. These were in comparison with a painless strength score of five on the contralateral shoulder. The rest of his examination was unremarkable.

## Management

**Figure 1:**
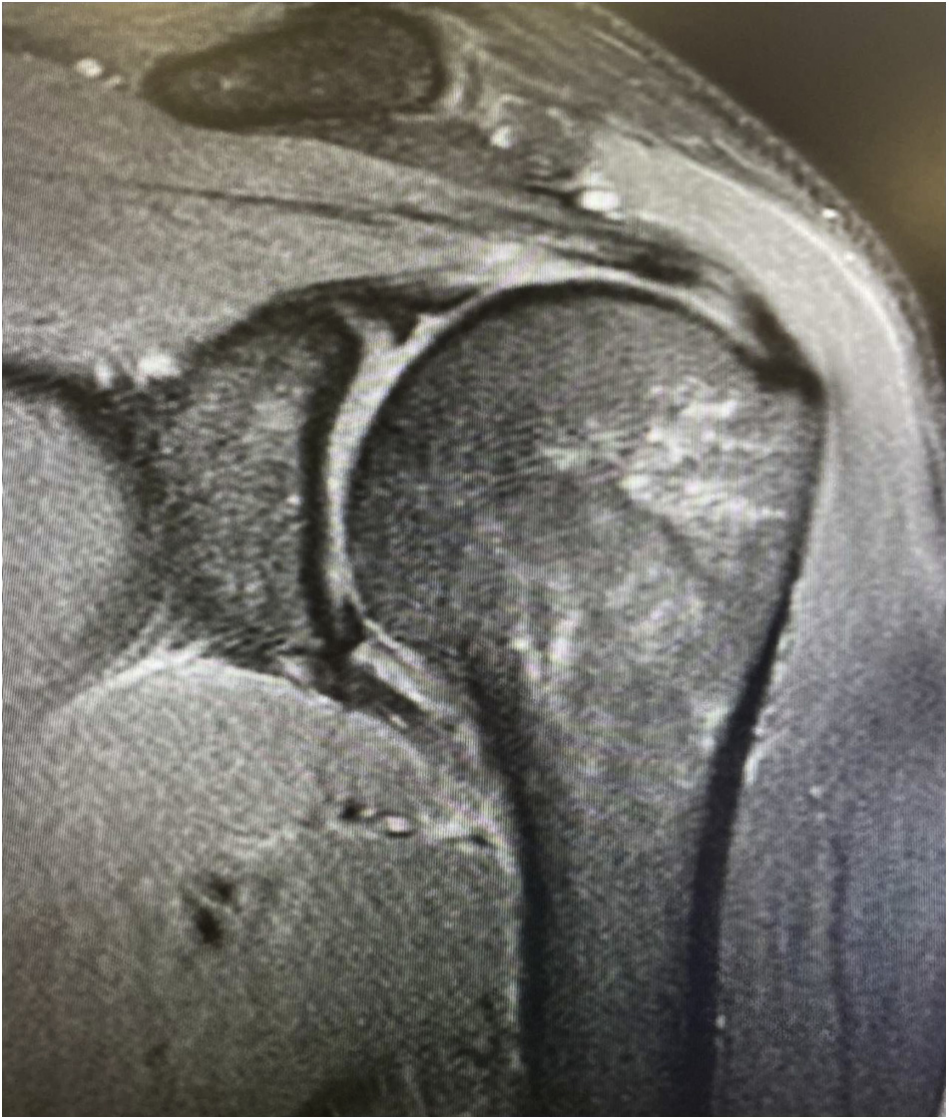
Our patient’s MRI significant for marginal tendonitis of the supraspinatus tendon with nonspecific subacromial edema. *MRI*, magnetic resonance imaging.

Consistent with our protocol, the patient received radiographs of the left shoulder, which did not show any evidence of acute trauma, superior humeral head migration, or osteoarthritis. Magnetic resonance imaging (MRI) was obtained, following clinical exclusion of other shoulder diagnoses, which showed marginal tendonitis of the supraspinatus tendon and slight edema in the subacromial area, which is nonspecific yet often associated with subacromial bursitis (Fig. 1). MRI findings in SIRVA cases typically present with fluid collections in the deltoid or overlying the rotator cuff tendons, bursitis, fluid “greater than typically seen” within the shoulder bursa, tendonitis, and rotator cuff tears.^1^ This presentation is unusual in comparison with normal postvaccination MRI findings that often include minor fluid accumulations of the deltoid and greater shoulder region.^1^

Based on the history, imaging, and physical examination findings, we made the diagnosis of SIRVA secondary to COVID-19 vaccine administration. After consultation with the patient, we decided that the next reasonable step would be to treat his shoulder symptoms with a subacromial corticosteroid.^7^ The American Academy of Orthopaedic Surgeons Patient Safety Committee recommends avoiding musculoskeletal corticosteroid injections for two weeks before and one week after COVID vaccine administration, but there is no direct evidence of the impact of corticosteroid injections on vaccine efficacy outside of that timeline. In a standard sterile fashion, the left subacromial space was injected with 20 mg triamcinolone with 9 cc of 1% Xylocaine without epinephrine.

Six days after the subacromial corticosteroid injection, the patient reported complete resolve of the pain stemming from postvaccine administration subacromial bursitis and rotator cuff tendinitis as a result of SIRVA. This included return of full active and passive shoulder range of motion, as well as painless strength scores of five in the empty can test and the external rotation test. As per the American Academy of Orthopaedic Surgeons, the postinjection pain phenomena should be acknowledged in which upward of 33% of patients may experience transient shoulder pain at the injection region up to four days after injection.

## Discussion

**Figure 2:**
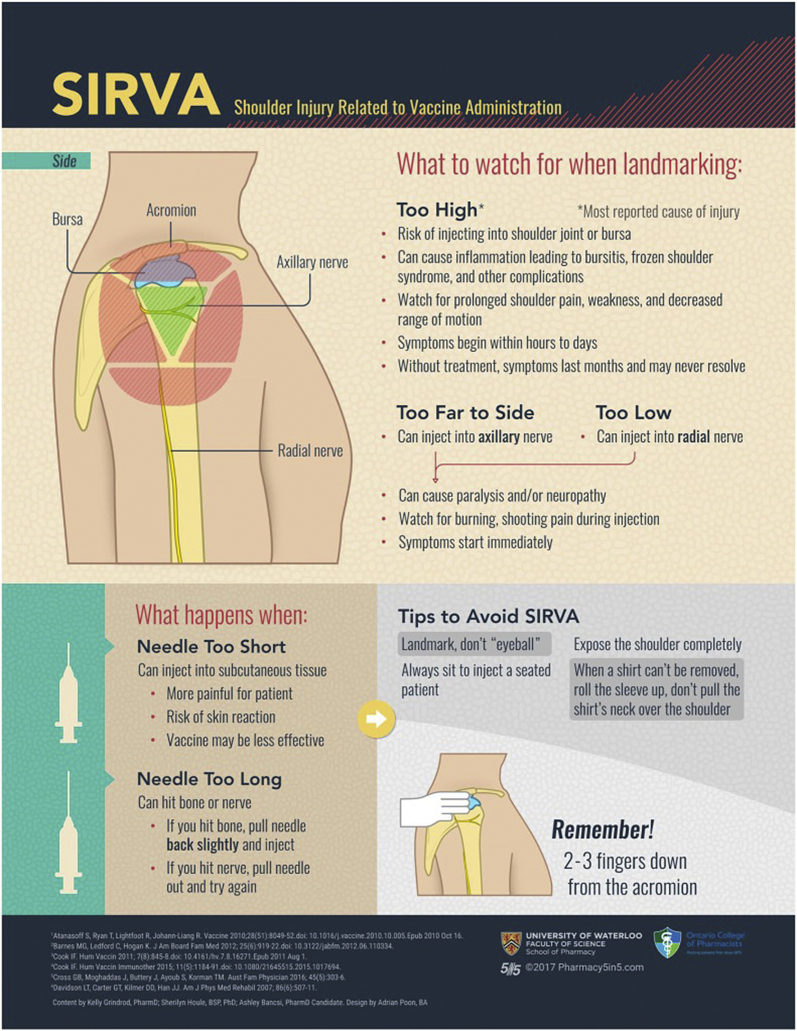
Appropriate vaccine administration technique to avoid SIRVA.^2^ (Content from Kelly Grindrod, PharmD; Sherilyn Houle, BSP, PhD; Ashley Bancsi, PharmD Candidate. Design by Adrian Poon, BA Copyright permission not necessary.)

After intramuscular vaccine administration, transient shoulder pain is one of the most frequent side effects experienced in the general population.^1^^,^^2^ Local inflammatory reactions in adults are common to present with any combination of erythema, pain, and induration at the local injection site after vaccine administration.^5^ Delayed deltoid soreness and dysfunction is uncommon and usually related to an inappropriate site of vaccine delivery.^9^ This reaction, known as SIRVA, is classically seen around 48 hours after vaccination and often results in severe local pain that transitions into weakness and overall decreased shoulder motion without neurological dysfunction.^8^ The proposed mechanism of this reaction imputes vaccine antigens and adjuvants to the peri-capsular and bursal spaces of the glenohumeral joint, thus provoking an inflammatory reaction in these areas.^1^^,^^3^ Alternatively, vaccine administration can also be performed too distal and be in the area of the traversing axillary nerve which can result in a local axillary neuritis and shoulder dysfunction related to local nerve irritation. SIRVA may also result from inappropriate needle length in relation to the patient’s overall weight. As per the most recent immunization guidelines, patients over 70 kg require a 1-inch needle and those weighing less require a 5/8-inch needle to properly administer an intramuscular vaccine.^2^^,^^3^ Diagnosing this condition can be challenging as other pathologies including subacromial bursitis, rotator cuff tendinitis, rotator cuff tear, or adhesive capsulitis all overlap significantly with the symptomatology of SIRVA.^1^^,^^4^ The most commonly implicated vaccines in SIRVA have been the influenza, pneumococcal, and diphtheria-tetanus-pertussis vaccines; however, there has not been any evidence to show that a specified vaccine is more likely to cause SIRVA, as the damage seems to arise from local immune reaction, rather than the antigens contained in the vaccination.^8^ According to the Center of Disease Control, over 154 million people in the United States have received at least one dose of the COVID-19 vaccine, and while shoulder pain has been the most commonly reported side effect reported with this novel mRNA vaccine, there have been very few reports of SIRVA specifically. After examining the current literature regarding COVID-19 vaccination reactions, there have been numerous studies relating vaccine administration and local injection site reactions primarily resulting in erythema, induration, and delayed tenderness, but few studies relating COVID-19 vaccination and SIRVA at this point.^4^ Although there is little literature regarding SIRVA in the case of COVID-19 mRNA vaccinations, there are many data capturing the effects of SIRVA resulting from various other vaccinations. Our patient very likely suffered shoulder pain, weakness, and motion deficits after his vaccination because of incorrect administration of the antigens and adjuvants into the subacromial space producing a robust inflammatory response. This is the one of the first reported cases of SIRVA after COVID-19 mRNA vaccination, but it is likely a very common occurrence which is being misdiagnosed simply as local inflammatory reaction. This is likely due to lack of knowledge surrounding the correct anatomic vaccine administration site as many health care workers are being asked to administer vaccines who may have never done so before. In addition, many other health care providers will be seeing this pathology that is unfamiliar with its presentation and pathoanatomy. Dissemination of this information is paramount for education of the health care providers involved in the vaccination effort as well as for the general public who may suffer avoidable disability and vaccine hesitancy related to this pathology. Correct administration technique and location is detailed in the following (Fig. 2).

## Conclusion

SIRVA is an ongoing issue that results from imprecise anatomic vaccine administration to the subacromial space instead of intramuscularly in the deltoid muscle bulk.^6^ Although still quite rare, it is important that instances of imprecise vaccine administration leading to SIRVA are documented to promote understanding of possible causes of obscure shoulder pain after vaccine administration.^3^ This will help prevent this avoidable side effect and mitigate vaccine hesitancy in the global population. In the case of our patient, the injury led to weeks of pain, weakness, and disability that went unresolved before corticosteroid injection therapy. Past literature suggests tendonitis/subacromial bursitis is associated in approximately half of documented SIRVA instances, much like that of our patient who received the novel COVID-19 vaccination from Moderna.^1^ Without intervention, various shoulder symptoms can continue months to years later without natural resolution.^9^ Overall, SIRVA is a preventable side effect with serious complications that can be addressed with the correct training and education to those administering vaccines,^2^ and it is a treatable condition if recognized by medical providers. By recognizing, treating, and preventing this pathology, medical professionals can directly help prevent harm to their patients and more effectively combat the COVID-19 pandemic.

## Disclaimers:

Funding: No funding was disclosed by the authors.

Conflicts of interest: The authors, their immediate families, and any research foundation with which they are affiliated have not received any financial payments or other benefits from any commercial entity related to the subject of this article.

Patient consent: Obtained.

## Supplementary Data

Patient Consent
