# Supplementary material for: Shoulder injury related to COVID-19 vaccine administration: a case report
Source: JSES Rev Rep Tech. 2021 Dec 4;2(2):178–81. doi: 10.1016/j.xrrt.2021.10.005 (PMC8641978; doi:10.1016/j.xrrt.2021.10.005)
Supplement: Patient Consent [file mmc1.docx]

We have received informed consent from the patient for this case report and for the manuscript text.
